# Behavioral and Psychological Symptoms in Alzheimer's Disease

**DOI:** 10.1155/2014/927804

**Published:** 2014-07-15

**Authors:** Xiao-Ling Li, Nan Hu, Meng-Shan Tan, Jin-Tai Yu, Lan Tan

**Affiliations:** ^1^Department of Neurology, Qingdao Municipal Hospital, School of Medicine, Qingdao University, No. 5 Donghai Middle Road, Qingdao 266071, China; ^2^Department of Neurology, Qingdao Municipal Hospital, College of Medicine and Pharmaceutics, Ocean University of China, Qingdao 266003, China

## Abstract

Neuropsychiatric symptoms (NPS) such as depression, apathy, aggression, and psychosis are now recognized as core features of Alzheimer's disease (AD), and there is a general consensus that greater symptom severity is predictive of faster cognitive decline, loss of independence, and even shorter survival. Whether these symptoms result from the same pathogenic processes responsible for cognitive decline or have unique etiologies independent of AD-associated neurodegeneration is unclear. Many structural and metabolic features of the AD brain are associated with individual neuropsychiatric symptoms or symptom clusters. In addition, many genes have been identified and confirmed that are associated with symptom risk in a few cases. However, there are no single genes strongly predictive of individual neuropsychiatric syndromes, while functional and structural brain changes unique to specific symptoms may reflect variability in progression of the same pathological processes. Unfortunately, treatment success for these psychiatric symptoms may be lower when comorbid with AD, underscoring the importance of future research on their pathobiology and treatment. This review summarizes some of the most salient aspects of NPS pathogenesis.

## 1. Introduction

**Figure 1:**
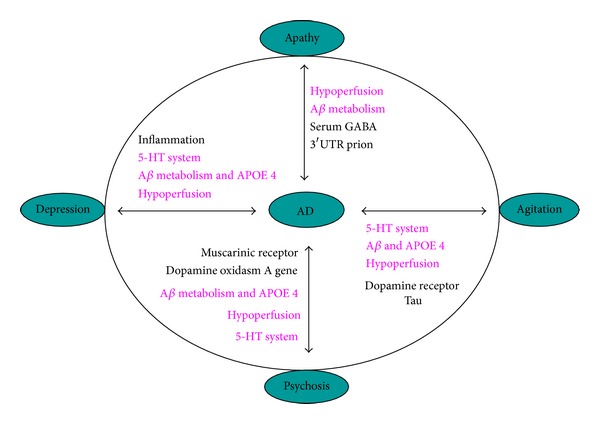
Possible mechanism linking the neuropsychiatric symptoms (NPS) with AD. NPS such as depression, apathy, aggression, and psychosis shared some pathogenic processes (in red color) with AD, while they also have their unique pathogenic processes.

The recent establishment of a professional interest area (PIA) within the International Society to Advance Alzheimer's Research and Treatment (ISTAART) devoted to the neuropsychiatric symptoms (NPS) of Alzheimer's is a sign of the emerging consensus among researchers and clinicians alike that these symptoms are major components of Alzheimer's disease (AD) and significant influences on both patient and caregiver quality of life (QOL) [1]. Indeed, neuropsychiatric symptoms such as apathy, depression, aggression, agitation, sleep disruption, and psychosis are now recognized as core symptoms of AD that are expressed to varying degrees throughout the course of the illness [2]. In addition to providing insight into AD pathology, specific neuropsychiatric and behavioral anomalies during the early prodromal phase of mild cognitive impairment (MCI) may have prognostic values. For example, late-life depression increases AD risk by 2-fold [3]. In this review, the major neuropsychiatric and behavioral symptoms of AD are reviewed with emphasis on how these symptoms may illuminate disease pathogenesis or provide prognostic information. Alzheimer's dementia is the end result of multiple pathogenic processes including aberrant amyloid processing [4, 5], changes in lipid metabolism due to apolipoprotein E (APOE) risk alleles [6, 7], tau hyperphosphorylation [8], protein misfolding and endoplasmic reticulum (ER) stress [9], vascular dysfunction [10], oxidative stress and mitochondrial dysfunction [11, 12], neurotrophic factor dysregulation [13], disrupted leptin signaling [14], fibrin clots [15], and processes mediated by a myriad of other AD-associated gene [16], and the pathogenic processes also occurred in major neuropsychiatric symptoms (Figure 1). It is likely that these processes target nonoverlapping neural networks, accounting for difference in disease progression and the variability in neuropsychiatric symptoms.

The neuropsychiatric symptoms and behavioral anomalies of AD have a significant impact on patient QOL and are thought to be predictive of eventual (or more severe) dementia [17, 18], more extensive neurodegeneration [19], loss of functional independence and institutionalization [20], and early death [21]. Thus, there is general agreement that these neuropsychiatric symptoms and behavioral anomalies are predictive of poor outcome, although symptom incidence, progression, and prognostic significance are highly variable across studies, possibly due to the different neuropsychiatric instruments used or clinical definitions. Moreover, despite recent identification of noninvasive biomarkers related to AD [22] and advances in imaging AD-associated plaques [23, 24], AD is still a diagnosis that can only be confirmed at autopsy, so most such studies relate neuropsychiatric symptoms to “presumed AD.” Another uncertainty is whether these neuropsychiatric and behavioral abnormalities such as depression and psychosis are etiologically similar in patients with and without AD or constitute clinical entities unique to AD.

## 2. Depression

Depression is a common comorbidity in AD, with prevalence estimates ranging from 25% to 74.9% in a group of recent studies [23–28]. This variability is likely due to the multitude of instruments used for diagnosis, including the DSM, Neuropsychiatric Inventory Depression subscale (NPI-D), and Geriatric Depression scale (GDS). Indeed, one study reported rates in the same cohort of 10.5% using the NPI-D (significant), 56.4% based on the NPI-D (any), 30% based on the Geriatric Depression scale (GDS), and 16% based on antidepressant usage [24]. A French Network on AD (REAL.FR) study following several hundred AD patients without depression or antidepressant use over 4 years reported an incidence of 17.45%/year [27]. Thus, about 40% of AD patients are expected to show symptoms of clinical depression within 5 years. Moreover, voluminous evidence indicates that AD with depression results in worse clinical outcome [20].

Based on studies in depressed non-AD populations, early studies on the pathophysiology of depression in AD focused on serotonergic transmission. One of the earliest studies reported an association between major depression in AD at baseline and 5-HT (2A) and 5-HT (2C) receptor polymorphisms, with CC carriers of the 5-HT (2A) C102 allele five times more likely than heterozygotes and 5-HT (2C) Ser allele carriers 12 times more likely than 5-HT (2C) Cys allele carriers to develop depression [29]. Moreover, reduced 5-HT (1A) receptor expression was specifically correlated with depressive symptoms [30]. In contrast, Pritchard and colleagues found no significant association between depression in AD and either the C allele/CC genotype of the T102C variant of 5HT (2A) or the cys23ser variant of 5HT (2C) receptor, although these alleles were associated with psychosis and aberrant motor behavior [31]. Moreover, no association was found between depression in AD and alleles of the serotonin transporter (SERT) [32]. Similarly, although SERT expression was reduced in the frontal cortex of AD patients, there was no difference in expression in patients with or without comorbid depression [33]. It is possible that serotonergic dysfunction may be heterogeneous among brain regions across patients, accounting for these differences in association. In addition to 5-HT signaling, elderly subjects destined to exhibit signs of major depression were more likely to harbor the GG genotype of the tumor necrosis factor (TNF)-alpha 308 (G/A) SNP variant, implicating inflammation in late-onset MD [34].

Early studies also examined the relationship between depression and molecules implicated in general AD pathology, particularly A*β* and APOE 4, the strongest risk allele for AD. Early onset depression was associated with a higher serum A*β*40/A*β*42 ratio [19], suggesting that depression may be associated with AD pathogenesis. One early small sample study found no association between APOE genotype and depression in AD [35], although subsequent studies have demonstrated that APOE genotype can modify the effects of other genes associated with the neuropsychiatric symptoms of AD (see below). A higher serum concentration of A*β* at baseline predicted both depression and AD over 5 years suggesting shared etiology [36]. Plasma GABA was positively correlated with depression and apathy scores on the NPI in AD patients [37].

In addition to gene association studies, the pathogenesis of AD has also been examined by various neuroimaging modalities, which have revealed morphological and metabolic signs of neurodegeneration in the AD brain specifically associated with depression. Compared to nondepressed AD patients, those with depression exhibited hypoperfusion in the left frontal lobe on single-photon emission computed tomography (SPECT) images [38] and reduced glucose metabolism in the dorsolateral prefrontal regions as revealed by (18)F-fluorodeoxyglucose PET [39]. Correlation analysis of brain SPECT and NPI score revealed a region in the left middle frontal gyrus (Brodmann area 9) specifically associated with depressive symptoms [40]. Depression in AD has also been associated with specific neurochemical changes; GDS scores but not agitation scores were correlated with choline/creatine ratio in left dorsolateral prefrontal cortex [41]. Cortical atrophy associated with depression was observed in wide regions of the prefrontal cortex and temporal cortex [42] and decreased gray matter volume in the left inferior temporal gyrus was confirmed in an independent study [43]. Depressed AD patients also exhibited greater white matter atrophy in frontal, temporal, and parietal lobes than AD patients without depressive symptoms [44]. One study also reported lesions in the caudate nucleus and lentiform nucleus of AD patients with late-onset depression [45]. Expansion of the lateral ventricles was also correlated with depression, general cognitive decline, and poor outcome [46]. Thus, depression is associated with both gray and white matter atrophy, particularly in specific regions of the prefrontal cortex.

However, it remains unclear if depression results from AD or conversely if geriatric depression is a risk factor for AD. In the first case, depression may be a psychological response to AD or result from the same pathogenic processes that lead to the other symptoms of AD (e.g., aberrant amyloid A*β* processing, tau hyperphosphorylation, etc.) [47]. Depression in AD is associated with accelerated cortical regression and white matter atrophy, particularly in frontal and temporal areas. It has been proposed that AD-associated degeneration may eventually damage regions involved in regulation of mood, a finding consistent with the high rates of depression in severe AD. Nonetheless, several genetic risk factors for major depression appear to increase the risk of depression in AD but not AD without depression, so the emergence of depression may not be entirely dependent on AD pathogenesis. For example, the tryptophan hydroxylase-1 (TPH1) A218C allele, monoamine oxidase A (MAOA) variable number tandem repeat (VNTR), and BDNF Val66Met allele were associated with depression in females with AD, with significantly increased likelihood of comorbid AD and depression in homozygous TPH1 A-allele and MAOA VNTR carriers [48]. In this same study, there was also a significant association between the chaperone FK506 binding protein 5 (FKBP5) rs1360780 SNP and depression in all AD patients. In addition, homozygous carriers of the rs10410544T allele of the SIRT2 gene (encoding an NAD-dependent deacetylase possibly involved in cell cycle regulation) may have reduced depression risk in AD [49]. Aside from the Val66Met allele of BDNF, the C allele of the SNP G11757C and the A allele of G196A were also more common in AD patients with depression [50]. One of the strongest associations with late-onset AD and depression is that with the transforming growth factor 1 (TGF-1) gene the CC genotype of the +10 T/C SNP was associated with AD and conferred a 5-fold increase in depression in AD as well as an increase in depression severity [51]. Finally, the presence of the APOE 4 allele increased depression in women with AD by 4-fold [52]. In contrast, another study reported that APOE 4 was associated with anxiety but not depression [53], while others have found no association between APOE 4 and neuropsychiatric symptoms [35].

Whether depression increases AD risk in premorbid or MCI patients is still a matter of debate. In an Italian study, newly diagnosed AD patients with persistent depression exhibited a greater cognitive decline over one year, and patients with incident depression demonstrated the greatest drop in cognitive function, while cognitive decline in cases with resolved depression was not different from nondepressed AD patients [54]. Late-onset depression does increase the risk of progression to MCI, but chronic depression was associated with only a modest increase in the risk of MCI-to-AD transition. Another Italian study reported that apathy but not depression was associated with MCI to AD transition [55]. In contrast, the Honolulu-Asia Aging Study using the Center for Epidemiological Studies depression scale (CES-D) reported that depression was an independent risk factor for cognitive decline in AD. Moreover, the effect was independent of pathological progression, such as increases in the number/density of neurofibrillary tangles (NTs), Lewy bodies, or ischemic lesions [56]. These differences in the reported prognostic value of depression may depend on diagnostic criteria; for example, the Vienna Transdanube Aging study did report an association with AD emergence over a 5-year period in 75-year-old individuals with no history of depression, but only 1 of 9 depression subsyndromes, “loss of interest,” was associated with AD risk [57]. Another report concluded that depression does appear to increase the risk of transition from MCI to dementia, but this effect was stronger for all-cause dementia and vascular dementia than AD [3] or exclusive to vascular dementia [58].

Regardless of this etiological relationship, it is clear that AD-associated depression markedly reduces cognitive capacity, QOL, and activities of daily function (ADF). Thus, treatment of depressive symptoms is expected to benefit AD patients. However, there have been relatively few controlled clinical trials on antidepressant therapy for depression in AD and clinical response is generally poor to modest [59–64]. The uncertain relationship between AD and depression undoubtedly arises in part from diagnostic uncertainty. As mentioned, AD is only confirmed at autopsy while estimates of depression vary marked depending on the instruments used. Furthermore, only certain depressive symptoms may be associated with AD [57]. In sum, depression may be a modest risk factor in premorbid patients [59–64] for additional reviews but when present it markedly reduces cognition, QOL, and ADL in AD patients.

## 3. Apathy

Apathy is defined by a cluster of motivational deficits such as loss of goal-directed cognition, action, and emotion [65]. Like other neuropsychiatric symptoms associated with AD, persistent apathy is predictive of more rapid cognitive decline compared to AD without apathy. Apathy and depression are often comorbid. In one relatively large cohort (255 patients), 47.9% of the study group had depression, 41.6% apathy, and 32.4% both, with smaller prevalence of depression and apathy alone (15.4% and 9.4% resp.) [23]. A similar pattern has been reported in other studies (e.g., 23% depression only, 23% depression + apathy, and 20% apathy only) [66, 67]. This frequent comorbidity suggests shared etiology. Indeed, like depression, apathy is generally associated with hypofrontality as well as serum GABA [37]. However, apathy was specifically correlated with hypometabolism in left orbitofrontal areas while depression was associated with hypometabolism in left dorsolateral prefrontal regions [39]. Scores on the frontal assessment battery (FAB) for executive function are decreased by both apathy and depression alone, but the largest decrease was observed in comorbid patients [68]. This hypofrontality has been correlated with AD-associated pathogenesis [68]. Retention of the (11C) Pittsburgh compound-B (PIB) under PET to reveal A*β* plaques was higher in the bilateral frontal cortex of patients with apathy as determined by the NPI apathy subscale than in AD patients without apathy, and apathy scores were positively correlated with PIB signal in bilateral frontal and right anterior cingulate cortices. No correlations were found between PIB and any other NPI subscale, including depression [68]. This same study found no correlation between apathy and morphometric changes by MRI. A larger scale study of the Alzheimer Disease Neuroimaging Initiative database found that cortical thinning in temporal cortex was associated with more severe apathy over time after correcting for multiple covariates such as sex, age, APOE genotype, premorbid intelligence, memory performance, processing speed, antidepressant use, and AD duration [69]. Studies of white matter atrophy in AD patients with apathy [70] have reported significantly reduced fractional anisotropy (FA) values in the genu of the corpus callosum, negative correlations between apathy scores and FA values in the left anterior and posterior cingulum, right superior longitudinal fasciculus, splenium, body and genu of the corpus callosum, and bilateral uncinate fasciculus [69] or right anterior cingulate cortex, right thalamus, and bilateral parietal cortex.

Possible genetic associations specific for apathy have not be investigated as extensively as possible depression-associated genes. In AD patients, T allele carriers of the 3′UTR prion-like protein were more likely to exhibit apathy, although scores were increased for many other NPI subscales [71]. While apathy is often comorbid with depression, apathy and depression may have different prognostic significance. Apathy but not depression was strongly associated with the transition from MCI to AD; MCI patients with amnestic-MCI and apathy were seven times more likely to progress to AD compared to amnestic-MCI patients without apathy after adjusting for covariates, including depression, while depression alone did not increase risk of transition [72]. Also distinct from depression, depression tends to stabilize during AD progression while apathy tends to increase [73].

## 4. Agitation and Aggression

Agitation and aggression are significant dangers both to patients and caregivers. Like other behavioral and neuropsychiatric abnormalities, rates of agitation and aggression correlate with cognitive decline, loss of independence, and other metrics of poor outcome [74]. Aggression and agitation are more common in male patients [75]. Among nursing home residences, the severity of cognitive decline/dementia was correlated with physical agitation and verbal aggression as measured by the Cohen-Mansfield Agitation Inventory (CMAI). The intensity of dementia disorders is associated most strongly with physical agitation and verbal aggression. Aggression- and (or) agitation-specific changes in neurochemistry and neuropathology have been observed. Based on the well-established correlation between frontal lobe serotonergic dysfunction and aggression, many studies have investigated the correlations between aggression presence/severity and 5-HT signaling molecules. Among the first such studies reported a significant association between aggression in AD and the more highly expressed serotonin transporter long variant (5-HTTPRL) [76]. Male AD patients with a history of agitation/aggression were also more likely to harbor the C allele of the 5-HT synthetic enzyme tryptophan hydroxylase A218C intronic polymorphism [77]. Aggressive patients also exhibited an altered 5-HT6 receptor to choline acetyltransferase (ChAT) ratio in frontal and temporal cortices [78] and reduced 5-HT1A binding in temporal cortex after controlling for dementia severity [79]. However, others have found a much more complex interaction among gender, dementia severity, aggression, and serotonergic function [80]. Serotonin transporter polymorphisms have also been linked to a combined aggressive/psychotic phenotype [81]. Polymorphisms of dopamine receptor alleles also confer complex associations among psychotic symptoms and aggressiveness. Psychosis and aggression were more frequent in DRD1 B2/B2 allele carriers [82]. In a subsequent study, carriers of the D1 B2 allele were more prone to aggression and (or) to experience hallucinations, while carriers of the DRD3 1 allele were more likely to experience delusions [83]. In fact, there is a strong correlation between psychosis and aggression. Delusions are among the strongest predictors of aggression [84], possibly because aggression is driven by specific delusions of persecution [85, 86].

The CMAI scores, but not GDS scores, were negatively correlated with the N-acetylaspartate (NAA) to creatine ratio in the left posterior cingulate gyrus [41], indicating neurochemical changes specific to the aggressive AD phenotype. Moreover, only agitation/irritability scores were correlated with A*β*42 accumulation [87] and only Behavioral Pathology in Alzheimer's Disease (Behave-AD) aggressiveness subscale scores correlated with serum BDNF [70] in AD and MCI patients. One study demonstrated a correlation between aggressive symptoms and the magnitude of locus coeruleus damage, suggesting a role for reduced norepinephrine or compensatory changes in adrenergic receptors in aggression [88]. In fact, a test of *β*_2_ receptor function, the growth hormone (GH) response to clonidine, was blunted in aggressive patients compared to nonaggressive AD patients [89]. Higher NPI Agitation and Aggression subscale scores were associated with greater atrophy in a large number of regions of interest in the frontal and cingulated cortices as well as the insula, amygdala, and hippocampus [90]. The phospho-tau to tau ratio was also higher postmortem in the frontal lobe of AD patients with aggression compared to those without [91]. Similarly, hippocampal NTs and hypoperfusion in the mesial temporal lobe [92] were associated with aggressive symptoms in AD. The frequency of the APOE 4 allele was also associated with aggression [93].

Neuroleptics and other antipsychotics may be modestly effective in treating aggression and agitation, but longer-term treatment is associated with a significant increase in adverse events [94].

## 5. Psychosis

Psychotic symptoms, including delusions and hallucinations, are obviously the most salient and serious neuropsychiatric symptoms associated with AD, also generally the least frequent symptoms during the early stages of AD [80]. Like depression, the emergence of psychotic symptoms predicts greater or more rapid cognitive decline [44, 95]. Hallucinations are among those noncognitive AD symptoms, also including cognitive impairment level, physical aggression, and depressive symptoms, strongly predictive of institutionalization [96]. A longitudinal study found that psychosis in AD (observed in 7.8% of patients) was associated with greater initial cognitive dysfunction, more accelerated cognitive decline, and greater mortality [97]. Like aggression, to which it is strongly correlated, psychosis is associated with difference in the 5-HT system, such as a higher frequency of the C allele and CC genotype of the T102C variant of 5HT (2A) receptors in patients with hallucinations and delusions [31]. There may also be an association between the SERT long form and psychosis in AD [96]. Moreover, psychosis is associated with CSF tau, suggesting more severe tauopathy in psychotic patients [98] and with greater intracellular accumulation of hyperphosphorylated tau [99]. The APOE 4 allele also increases psychosis risk [100]. In general, AD-associated psychosis follows the severity of AD-associated neurodegeneration and cognitive dysfunction [101]. ^18^F-Fluorodeoxyglucose positron emission tomography revealed reduced metabolic activity in the right lateral frontal cortex, orbitofrontal cortex, and bilateral temporal cortex in patients with delusions, overlapping with areas associated with loss of memory and insight [102]. Atrophy of the supramarginal cortex of the parietal lobe was predictive of increasing hallucinations over time. Active psychosis is associated with hypofrontality, particularly in orbitofrontal regions [102], and thus is strongly correlated with disruption in executive function, particularly working memory [103]. Another study found lateral frontal, lateral parietal, and anterior cingulate atrophy in AD patients with psychosis, with the lateral frontal region most severely degenerated [104]. Individual delusions may be associated with specific abnormalities in neural processing as evidence by PET imaging [105], with delusions of persecution associated with hypoperfusion in the precuneus and hyperperfusion in the insula and thalamus. Other delusional forms are associated with distinct changes in perfusion, metabolism, receptor binding, and structural alternations [106]. Persecutory delusions occur early during the progression of AD and associate with disruption of frontostriatal circuits [107]. One study found delusions in 27.4% of AD patients, with paranoid delusions being the most common (60.3%), followed by misidentification delusions (19.0%), and then mixed delusions (17.5%), the latter appearing later and associated with greater cognitive impairment [108]. Psychotic patients show greater A*β*1-42 at autopsy [109]. A recent genome wide association study identified an intergenic region on chromosome 4 (rs753129), SNPs upstream of SLC2A9 (rs6834555), and within the neuronal Ca^2+^-sensor (NCS) proteins VSNL1 (rs4038131) as promising regions for specific associations with psychosis [110]. Note, however, that VSNLs are associated with A*β* and tau and so may reflect the overall severity of AD neurodegeneration rather than psychosis per se. Other possible associations specific to AD-associated psychosis include that with the dopamine oxidase A (DOA) gene and muscarinic receptors in orbital frontal cortex [110].

## 6. Conclusion

The neuropsychiatric symptoms in the early stages of AD are predictive of more rapid deterioration of cognitive function. These symptoms may simply reflect more rapid and extensive neuronal death caused by the myriad of primary and secondary degenerative processes associated with AD. In this case, the appearance of different neuropsychiatric symptoms reflects variability in the progression of neurodegeneration across neural systems. There are genes associated with specific symptoms or symptom clusters, but none appear to exert strong and specific effects. Moreover, many of these association studies have not been followed up, suggesting some publication bias for positive results. Regardless of the dependence of these symptoms on AD pathogenesis and cognitive decline, the deleterious effect of these symptoms on both patient and caregiver quality of life warrant further studies on more effective treatments.

## References

[1] Geda YE, Schneider LS, Gitlin LN (2013). Neuropsychiatric symptoms in Alzheimer's disease: past progress and anticipation of the future. *Alzheimer's & Dementia*.

[2] Lyketsos CG, Carrillo MC, Ryan JM (2011). Neuropsychiatric symptoms in Alzheimer's disease. *Alzheimer's & Dementia*.

[3] Barnes DE, Yaffe K, Byers AL, McCormick M, Schaefer C, Whitmer RA (2012). Midlife vs late-life depressive symptoms and risk of dementia: differential effects for Alzheimer disease and vascular dementia. *Archives of General Psychiatry*.

[4] Gilbert BJ (2013). The role of amyloid *β* in the pathogenesis of Alzheimer's disease. *Journal of Clinical Pathology*.

[5] Nalivaeva NN, Turner AJ (2013). The amyloid precursor protein: a biochemical enigma in brain development, function and disease. *FEBS Letters*.

[6] Liu C, Kanekiyo T, Xu H, Bu G (2013). Apolipoprotein e and Alzheimer disease: risk, mechanisms and therapy. *Nature Reviews Neurology*.

[7] Reitz C (2013). Dyslipidemia and the risk of Alzheimer's disease. *Current Atherosclerosis Reports*.

[8] Duan Y, Dong S, Gu F, Hu Y, Zhao Z (2012). Advances in the pathogenesis of Alzheimer's disease: focusing on tau-mediated neurodegeneration. *Translational Neurodegeneration*.

[9] Cornejo VH, Hetz C (2013). The unfolded protein response in Alzheimer's disease. *Seminars in Immunopathology*.

[10] Kelleher RJ, Soiza RL (2013). Evidence of endothelial dysfunction in the development of Alzheimer's disease: is Alzheimer's a vascular disorder?. *The American Journal of Cardiovascular Disease*.

[11] Caldeira GL, Ferreira IL, Rego AC (2013). Impaired transcription in Alzheimer's disease: key role in mitochondrial dysfunction and oxidative stress. *Journal of Alzheimer's Disease*.

[12] Swomley AM, Forster S, Keeney JT (2013). Abeta, oxidative stress in Alzheimer disease: evidence based on proteomics studies. *Biochimica et Biophysica Acta*.

[13] Sopova K, Gatsiou K, Stellos K, Laske C (2014). Dysregulation of neurotrophic and haematopoietic growth factors in Alzheimer's disease: from pathophysiology to novel treatment strategies. *Current Alzheimer Research*.

[14] Marwarha G, Ghribi O (2012). Leptin signaling and Alzheimer's disease. *The American Journal of Neurodegenerative Disease*.

[15] Lipinski B, Pretorius E (2013). The role of iron-induced fibrin in the pathogenesis of Alzheimer's disease and the protective role of magnesium. *Frontiers in Human Neuroscience*.

[16] Ramanan VK, Saykin AJ (2013). Pathways to neurodegeneration: mechanistic insights from GWAS in Alzheimer's disease, Parkinson's disease, and related disorders. *The American Journal of Neurodegenerative Disease*.

[17] Peters ME, Rosenberg PB, Steinberg M (2013). Neuropsychiatric symptoms as risk factors for progression from CIND to dementia: the cache county study. *The American Journal of Geriatric Psychiatry*.

[18] Diniz BS, Butters MA, Albert SM, Dew MA, Reynolds CF (2013). Late-life depression and risk of vascular dementia and Alzheimer's disease: systematic review and meta-analysis of community-based cohort studies. *The British Journal of Psychiatry*.

[19] Namekawa Y, Baba H, Maeshima H (2013). Heterogeneity of elderly depression: Increased risk of Alzheimer's disease and A*β* protein metabolism. *Progress in Neuro-Psychopharmacology and Biological Psychiatry*.

[20] Zahodne LB, Ornstein K, Cosentino S, Devanand DP, Stern Y (2013). Longitudinal relationships between Alzheimer disease progression and psychosis, depressed mood, and agitation/aggression. *The American Journal of Geriatric Psychiatry*.

[21] Vilalta-Franch J, López-Pousa S, Calvó-Perxas L, Garre-Olmo J (2013). Psychosis of Alzheimer disease: prevalence, incidence, persistence, risk factors, and mortality. *The American Journal of Geriatric Psychiatry*.

[22] Rosén C, Hansson O, Blennow K, Zetterberg H (2013). Fluid biomarkers in Alzheimer's disease—current concepts. *Molecular Neurodegeneration*.

[23] Benoit M, Berrut G, Doussaint J (2012). Apathy and depression in mild Alzheimer's disease: a cross-sectional study using diagnostic criteria. *Journal of Alzheimer's Disease*.

[24] Kwak YT, Yang Y, Pyo SJ, Koo MS (2013). Clinical characteristics according to depression screening tools in patients with Alzheimer's disease: view from self, caregiver-reported and drug-intervention pattern. *Geriatrics & Gerontology International*.

[25] Borroni B, Archetti S, Costanzi C (2009). Role of BDNF Val66Met functional polymorphism in Alzheimer's disease-related depression. *Neurobiology of Aging*.

[26] Usman S, Chaudhary HR, Asif A, Yahya MI (2010). Severity and risk factors of depression in Alzheimer's disease. *Journal of the College of Physicians and Surgeons Pakistan*.

[27] Arbus C, Gardette V, Cantet CE (2011). Incidence and predictive factors of depressive symptoms in Alzheimer's disease: the REAL.FR study. *Journal of Nutrition, Health and Aging*.

[28] Van der Mussele S, Bekelaar K, Le Bastard N (2013). Prevalence and associated behavioral symptoms of depression in mild cognitive impairment and dementia due to Alzheimer's disease. *International Journal of Geriatric Psychiatry*.

[29] Holmes C, Arranz M, Collier D, Powell J, Lovestone S (2003). Depression in Alzheimer's disease: The effect of serotonin receptor gene variation. *The American Journal of Medical Genetics—Neuropsychiatric Genetics*.

[30] Lai MKP, Tsang SW, Esiri MM, Francis PT, Wong PT, Chen CP (2011). Differential involvement of hippocampal serotonin1A receptors and re-uptake sites in non-cognitive behaviors of Alzheimer's disease. *Psychopharmacology*.

[31] Pritchard AL, Harris J, Pritchard CW (2008). Role of 5HT_2A_ and 5HT_2C_ polymorphisms in behavioural and psychological symptoms of Alzheimer's disease. *Neurobiology of Aging*.

[32] Pritchard AL, Pritchard CW, Bentham P, Lendon CL (2007). Role of serotonin transporter polymorphisms in the behavioural and psychological symptoms in probable Alzheimer disease patients. *Dementia and Geriatric Cognitive Disorders*.

[33] Thomas AJ, Hendriksen M, Piggott M (2006). A study of the serotonin transporter in the prefrontal cortex in late-life depression and Alzheimer's disease with and without depression. *Neuropathology and Applied Neurobiology*.

[34] Cerri AP, Arosio B, Viazzoli C, Confalonieri R, Teruzzi F, Annoni G (2009). -308(G/A) TNF-alpha gene polymorphism and risk of depression late in the life. *Archives of Gerontology and Geriatrics*.

[35] Liu CY, Hong CJ, Liu TY (2002). Lack of association between the apolipoprotein E genotype and depression in Alzheimer's disease. *Journal of Geriatric Psychiatry and Neurology*.

[36] Blasko I, Kemmler G, Jungwirth S (2010). Plasma amyloid beta-42 independently predicts both late-onset depression and Alzheimer disease. *The American Journal of Geriatric Psychiatry*.

[37] Lanctôt KL, Herrmann N, Rothenburg L, Eryavec G (2007). Behavioral correlates of GABAergic disruption in Alzheimer's disease. *International Psychogeriatrics*.

[38] Kataoka K, Hashimoto H, Kawabe J (2010). Frontal hypoperfusion in depressed patients with dementia of Alzheimer type demonstrated on 3DSRT. *Psychiatry and Clinical Neurosciences*.

[39] Holthoff VA, Beuthien-Baumann B, Kalbe E (2005). Regional cerebral metabolism in early Alzheimer's disease with clinically significant apathy or depression. *Biological Psychiatry*.

[40] Terada S, Oshima E, Sato S (2014). Depressive symptoms and regional cerebral blood flow in Alzheimer's disease. *Psychiatry Research*.

[41] Tsai CF, Hung CW, Lirng JF, Wang SJ, Fuh JL (2013). Differences in brain metabolism associated with agitation and depression in Alzheimer's disease. *East Asian Archives of Psychiatry*.

[42] Lebedev AV, Beyer MK, Fritze F, Westman E, Ballard C, Aarsland D (2014). Cortical changes associated with depression and antidepressant use in Alzheimer and Lewy body dementia: an MRI surface-based morphometric study. *The American Journal of Geriatric Psychiatry*.

[43] Son JH, Han DH, Min KJ, Kee BS (2013). Correlation between gray matter volume in the temporal lobe and depressive symptoms in patients with Alzheimer's disease. *Neuroscience Letters*.

[44] Lee GJ, Lu PH, Hua X (2012). Depressive symptoms in mild cognitive impairment predict greater atrophy in alzheimer's disease-related regions. *Biological Psychiatry*.

[45] Brommelhoff JA, Spann BM, Go JL, MacK WJ, Gatz M (2011). Striatal hypodensities, not white matter hypodensities on CT, Are Associated with Late-Onset Depression in Alzheimer's Disease. *Journal of Aging Research*.

[46] Chou Y-Y, Leporé N, Saharan P (2010). Ventricular maps in 804 ADNI subjects: correlations with CSF biomarkers and clinical decline. *Neurobiology of Aging*.

[47] Mintzer J, O'Neill C (2011). Depression in Alzheimer's disease: consequence or contributing factor?. *Expert Review of Neurotherapeutics*.

[48] Arlt S, Demiralay C, Tharun B (2013). Genetic risk factors for depression in Alzheimer`s disease patients. *Current Alzheimer Research*.

[49] Porcelli S, Salfi R, Politis A (2013). Association between Sirtuin 2 gene rs10410544 polymorphism and depression in Alzheimer's disease in two independent European samples. *Journal of Neural Transmission*.

[50] Zhang L, Fang Y, Zeng Z (2011). BDNF gene polymorphisms are associated with Alzheimer's disease-related depression and antidepressant response. *Journal of Alzheimer's Disease*.

[51] Caraci F, Bosco P, Signorelli M (2012). The CC genotype of transforming growth factor-*β*1 increases the risk of late-onset Alzheimer's disease and is associated with AD-related depression. *European Neuropsychopharmacology*.

[52] Delano-Wood L, Houston WS, Emond JA (2008). APOE genotype predicts depression in women with Alzheimer's disease: a retrospective study. *International Journal of Geriatric Psychiatry*.

[53] Michels A, Multhammer M, Zintl M, Mendoza MC, Klünemann H (2012). Association of apolipoprotein E *ε*4 (ApoE *ε*4) homozygosity with psychiatric behavioral symptoms. *Journal of Alzheimer's Disease*.

[54] Spalletta G, Caltagirone C, Girardi P, Gianni W, Casini AR, Palmer K (2012). The role of persistent and incident major depression on rate of cognitive deterioration in newly diagnosed Alzheimer's disease patients. *Psychiatry Research*.

[55] Palmer K, Di Iulio F, Varsi AE (2010). Neuropsychiatric predictors of progression from amnestic—Mild cognitive impairment to Alzheimer's disease: the role of depression and apathy. *Journal of Alzheimer's Disease*.

[56] Royall DR, Palmer RF (2013). Alzheimer's disease pathology does not mediate the association between depressive symptoms and subsequent cognitive decline. *Alzheimer's and Dementia*.

[57] Mossaheb N, Zehetmayer S, Jungwirth S (2012). Are specific symptoms of depression predictive of Alzheimer's dementia?. *Journal of Clinical Psychiatry*.

[58] Lenoir H, Dufouil C, Auriacombe S (2011). Depression history, depressive symptoms, and incident dementia: the 3C study. *Journal of Alzheimer's Disease*.

[59] Even C, Weintraub D (2010). Case for and against specificity of depression in Alzheimer's disease. *Psychiatry and Clinical Neurosciences*.

[60] Aznar S, Knudsen GM (2011). Depression and alzheimer's disease: is stress the initiating factor in a common neuropathological cascade?. *Journal of Alzheimer's Disease*.

[61] Caraci F, Copani A, Nicoletti F, Drago F (2010). Depression and Alzheimer's disease: neurobiological links and common pharmacological targets. *European Journal of Pharmacology*.

[62] Modrego PJ (2010). Depression in Alzheimer's disease. Pathophysiology, diagnosis, and treatment. *Journal of Alzheimer's Disease*.

[63] Sierksma AS, van den Hove DL, Steinbusch HW, Prickaerts J (2010). Major depression, cognitive dysfunction and Alzheimer's disease: is there a link?. *European Journal of Pharmacology*.

[64] Wuwongse S, Chang RC, Law ACK (2010). The putative neurodegenerative links between depression and Alzheimer's disease. *Progress in Neurobiology*.

[65] Ota M, Sato N, Nakata Y, Arima K, Uno M (2012). Relationship between apathy and diffusion tensor imaging metrics of the brain in Alzheimers disease. *International Journal of Geriatric Psychiatry*.

[66] Grossi D, Santangelo G, Barbarulo AM (2013). Apathy and related executive syndromes in dementia associated with Parkinson's disease and in Alzheimer's disease. *Behavioural Neurology*.

[67] Nakaaki S, Murata Y, Sato J (2008). Association between apathy/depression and executive function in patients with Alzheimer's disease. *International Psychogeriatrics*.

[68] Mori T, Shimada H, Shinotoh H (2014). Apathy correlates with prefrontal amyloid beta deposition in Alzheimer's disease. *Journal of Neurology, Neurosurgery & Psychiatry*.

[69] Hahn C, Lim H, Won WY, Ahn KJ, Jung W, Lee CU (2013). Apathy and white matter integrity in Alzheimer's Disease: a whole brain analysis with tract-based spatial statistics. *PLoS ONE*.

[70] Donovan NJ, Wadsworth LP, Lorius N (2013). Regional cortical thinning predicts worsening apathy and hallucinations across the Alzheimer disease spectrum. *American Journal of Geriatric Psychiatry*.

[71] Flirski M, Sieruta M, Golańska E, Kłoszewska I, Liberski PP, Sobów T (2012). PRND 3′UTR polymorphism may be associated with behavioral disturbances in Alzheimer disease. *Prion*.

[72] Richard E, Schmand B, Eikelenboom P (2012). Symptoms of apathy are associated with progression from mild cognitive impairment to Alzheimer's disease in non-depressed subjects for the Alzheimer's disease neuroimaging initiative. *Dementia and Geriatric Cognitive Disorders*.

[73] Gonfrier S, Andrieu S, Renaud D, Vellas B, Robert P (2012). Course of neuropsychiatric symptoms during a 4-year follow up in the REAL-FR cohort. *Journal of Nutrition, Health and Aging*.

[74] Bidzan L, Bidzan M, Pachalska M (2012). Aggressive and impulsive behavior in Alzheimer's disease and progression of dementia. *Medical Science Monitor*.

[75] Kitamura T, Kitamura M, Hino S, Tanaka N, Kurata K (2012). Gender differences in clinical manifestations and outcomes among hospitalized patients with behavioral and psychological symptoms of dementia. *Journal of Clinical Psychiatry*.

[76] Sukonick DL, Pollock BG, Sweet RA (2001). The 5-HTTPR^∗^S/^∗^L polymorphism and aggressive behavior in Alzheimer disease. *Archives of Neurology*.

[77] Craig D, Hart DJ, Carson R, McIlroy SP, Passmore AP (2004). Allelic variation at the A218C tryptophan hydroxylase polymorphism influences agitation and aggression in Alzheimer's disease. *Neuroscience Letters*.

[78] Garcia-Alloza M, Hirst WD, Chen CPL, Lasheras B, Francis PT, Ramírez MJ (2004). Differential involvement of 5-HT(1B/1D) and 5-HT6 receptors in cognitive and non-cognitive symptoms in Alzheimer's disease. *Neuropsychopharmacology*.

[79] Lai MKP, Tsang SWY, Francis PT (2003). Reduced serotonin 5-HT1A receptor binding in the temporal cortex correlates with aggressive behavior in Alzheimer disease. *Brain Research*.

[80] Lanctôt KL, Herrmann N, Eryavec G, van Reekum R, Reed K, Naranjo CA (2002). Central serotonergic activity is related to the aggressive behaviors of Alzheimer's disease. *Neuropsychopharmacology*.

[81] Sweet RA, Pollock BG, Sukonick DL (2001). The 5-HTTPR polymorphism confers liability to a combined phenotype of psychotic and aggressive behavior in Alzheimer disease. *International Psychogeriatrics*.

[82] Sweet RA, Nimgaonkar VL, Kamboh ML, Lopez OL, Zhang F, Dekosky ST (1998). Dopamine receptor genetic variation, psychosis, and aggression in Alzheimer disease. *Archives of Neurology*.

[83] Holmes C, Smith H, Ganderton R (2001). Psychosis and aggression in Alzheimer's disease: the effect of dopamine receptor gene variation. *Journal of Neurology Neurosurgery and Psychiatry*.

[84] Gormley N, Rizwan MR, Lovestone S (1998). Clinical predictors of aggressive behaviour in Alzheimer's disease. *International Journal of Geriatric Psychiatry*.

[85] Gilley DW, Wilson RS, Beckett LA, Evans DA (1997). Psychotic symptoms and physically aggressive behavior in Alzheimer's disease. *Journal of the American Geriatrics Society*.

[86] Eustace A, Kidd N, Greene E (2001). Verbal aggression in Alzheimer's disease. Clinical, functional and neuropsychological correlates. *International Journal of Geriatric Psychiatry*.

[87] Nagata T, Kobayashi N, Shinagawa S, Yamada H, Kondo K, Nakayama K (2013). Plasma BDNF levels are correlated with aggressiveness in patients with amnestic mild cognitive impairment or Alzheimer disease. *Journal of Neural Transmission*.

[88] Matthews KL, Chen CPL, Esiri MM, Keene J, Minger SL, Francis PT (2002). Noradrenergic changes, aggressive behavior, and cognition in patients with dementia. *Biological Psychiatry*.

[89] Herrmann N, Lanctôt KL, Eryavec G, van Reekum R, Khan LR (2004). Growth hormone response to clonidine predicts aggression in Alzheimer's disease. *Psychoneuroendocrinology*.

[90] Trzepacz PT, Yu P, Bhamidipati PK (2013). Frontolimbic atrophy is associated with agitation and aggression in mild cognitive impairment and Alzheimer's disease. *Alzheimer's and Dementia*.

[91] Guadagna S, Esiri MM, Williams RJ, Francis PT (2012). Tau phosphorylation in human brain: relationship to behavioral disturbance in dementia. *Neurobiology of Aging*.

[92] Lanctôt KL, Herrmann N, Nadkarni NK, Leibovitch FS, Caldwell CB, Black SE (2004). Medial temporal hypoperfusion and aggression in Alzheimer disease. *Archives of Neurology*.

[93] Craig D, Hart DJ, McCool K, McIlroy SP, Passmore AP (2004). Apolipoprotein E e4 allele influences aggressive behaviour in Alzheimer's disease. *Journal of Neurology, Neurosurgery and Psychiatry*.

[94] Ballard C, Corbett A (2013). Agitation and aggression in people with Alzheimer's disease. *Current Opinion in Psychiatry*.

[95] Wilkosz PA, Seltman HJ, Devlin B (2010). Trajectories of cognitive decline in Alzheimer's disease. *International Psychogeriatrics*.

[96] Creese B, Ballard C, Jones E (2013). Cognitive impairment in studies of 5HTTLPR and psychosis in alzheimer's disease: a systematic review. *Dementia and Geriatric Cognitive Disorders*.

[97] Gilley DW, Bienias JL, Wilson RS, Bennett DA, Beck TL, Evans DA (2004). Influence of behavioral symptoms on rates of institutionalization for persons with Alzheimer's disease. *Psychological Medicine*.

[98] Koppel J, Sunday S, Buthorn J, Goldberg T, Davies P, Greenwald B (2013). Elevated CSF Tau is associated with psychosis in Alzheimer's disease. *The American Journal of Psychiatry*.

[99] Murray PS, Kirkwood CM, Gray MC (2014). Hyperphosphorylated Tau is elevated in Alzheimer's disease with psychosis. *Journal of Alzheimer’s Disease*.

[100] Zdanys KF, Kleiman TG, MacAvoy MG (2007). Apolipoprotein E _*ε*_4 allele increases risk for psychotic symptoms in Alzheimer's disease. *Neuropsychopharmacology*.

[101] Sultzer DL, Leskin LP, Melrose RJ (2013). Neurobiology of delusions, memory, and insight in alzheimer disease. *The American Journal of Geriatric Psychiatry*.

[102] Koppel J, Sunday S, Goldberg TE, Davies P, Christen E, Greenwald BS (2013). Psychosis in Alzheimer's disease is associated with frontal metabolic impairment and accelerated decline in working memory: findings from the Alzheimer's disease neuroimaging initiative. *The American Journal of Geriatric Psychiatry*.

[103] Koppel J, Goldberg TE, Gordon ML (2012). Relationships between behavioral syndromes and cognitive domains in alzheimer disease: the impact of mood and psychosis. *The American Journal of Geriatric Psychiatry*.

[104] Rafii MS, Taylor CS, Kim HT (2014). Neuropsychiatric symptoms and regional neocortical atrophy in mild cognitive impairment and Alzheimer's disease. *American Journal of Alzheimer's Disease & Other Dementias*.

[105] Nomura K, Kazui H, Wada T (2012). Classification of delusions in Alzheimer's disease and their neural correlates. *Psychogeriatrics*.

[106] Ismail Z, Nguyen M, Fischer CE, Schweizer TA, Mulsant BH (2012). Neuroimaging of delusions in Alzheimer's disease. *Psychiatry Research—Neuroimaging*.

[107] Reeves SJ, Gould RL, Powell JF, Howard RJ (2012). Origins of delusions in Alzheimer's disease. *Neuroscience & Biobehavioral Reviews*.

[108] Kwak YT, Yang Y, Kwak S, Koo M (2013). Delusions of Korean patients with Alzheimer's disease: Study of drug-naïve patients. *Geriatrics and Gerontology International*.

[109] Murray PS, Kirkwood CM, Gray MC (2012). *β*-Amyloid 42/40 ratio and kalirin expression in Alzheimer disease with psychosis. *Neurobiology of Aging*.

[110] Di Maria E, Bonvicini C, Bonomini C, Alberici A, Zanetti O, Gennarelli M (2009). Genetic variation in the G720/G30 gene locus (DAOA) influences the occurrence of psychotic symptoms in patients with Alzheimer's disease. *Journal of Alzheimer's Disease*.

